# Gabapentin Inhibits Protein Kinase C Epsilon Translocation in Cultured Sensory Neurons with Additive Effects When Coapplied with Paracetamol (Acetaminophen)

**DOI:** 10.1155/2017/3595903

**Published:** 2017-02-16

**Authors:** Vittorio Vellani, Chiara Giacomoni

**Affiliations:** ^1^Dipartimento di Scienze Biomediche, Metaboliche e Neuroscienze, Università di Modena e Reggio Emilia, Via Campi 287, 41125 Modena, Italy; ^2^Dipartimento di Economia, Scienze e Diritto, Università degli Studi della Repubblica di San Marino, Salita alla Rocca 44, 47890 San Marino (Città), San Marino

## Abstract

Gabapentin is a well-established anticonvulsant drug which is also effective for the treatment of neuropathic pain. Although the exact mechanism leading to relief of allodynia and hyperalgesia caused by neuropathy is not known, the blocking effect of gabapentin on voltage-dependent calcium channels has been proposed to be involved. In order to further evaluate its analgesic mechanisms, we tested the efficacy of gabapentin on protein kinase C epsilon (PKC*ε*) translocation in cultured peripheral neurons isolated from rat dorsal root ganglia (DRGs). We found that gabapentin significantly reduced PKC*ε* translocation induced by the pronociceptive peptides bradykinin and prokineticin 2, involved in both inflammatory and chronic pain. We recently showed that paracetamol (acetaminophen), a very commonly used analgesic drug, also produces inhibition of PKC*ε*. We tested the effect of the combined use of paracetamol and gabapentin, and we found that the inhibition of translocation adds up. Our study provides a novel mechanism of action for gabapentin in sensory neurons and suggests a mechanism of action for the combined use of paracetamol and gabapentin, which has recently been shown to be effective, with a cumulative behavior, in the control of postoperative pain in human patients.

## 1. Introduction

Gabapentin, a structural analogue of the inhibitory neurotransmitter gamma-aminobutyric acid, was originally developed in the early nineties as a third-generation antiepileptic drug. Besides epilepsy, gabapentin has been reported to be effective for the treatment of other pathological states such psychiatric and movement disorders, alcohol addiction, and restless leg syndrome [1]. In particular, gabapentin has been shown to have central and peripheral antinociceptive activity in several painful states with neuropathic-related features [2], including the inflammatory hyperalgesia and allodynia which develop after surgical procedures and may induce central sensitization and eventually neuropathic pain [3].

Paracetamol is a nonprescription analgesic/antipyretic drug with little anti-inflammatory activity and partial inhibition of COX-1 and COX-2 [4, 5], of widespread use both as a self-administered medication and in the clinical practice. The combination of gabapentin and paracetamol has been successfully used in postoperative patients, showing additive effect on morphine sparing [6]. Little is known about how gabapentin and paracetamol combine their actions to produce analgesia, but the additive effect suggests that convergence on related mechanisms may be hypothesized.

The epsilon isoform of protein kinase C (PKC*ε*) is a very important noxious effector [7, 8] currently regarded as a novel target for therapeutic intervention. PKC*ε* is involved in both inflammatory and neuropathic pain states [9–12] caused by diabetes [13] and alcohol abuse [14] and during chemotherapy [15]. Translocation is necessary to allow phosporylation by PKC*ε* of specific cellular targets located in the plasma membrane in nociceptive neurons [16, 17], including TRPV1 (Transient Receptor Potential Vanilloid 1) ion channels [18] and several other targets involved in nociceptor sensitization and excitability. Inhibition of translocation in peripheral fibers will prevent phosphorylation activity and result in antinociceptive effects in sensory neurons. Indeed, local, intradermal injection of PKC*ε* specific inhibitors at the site of nociceptive testing significantly inhibited pain in several models of peripheral hyperalgesia [13–15]. We recently showed that nimesulide, a nonsteroidal anti-inflammatory drug (NSAID), and paracetamol inhibit translocation of PKC*ε* induced in cultured sensory neurons by bradykinin and thrombin, inflammatory mediators that sensitize TRPV1 to heat stimuli and to capsaicin [18–21]. We therefore proposed that nimesulide and paracetamol may exert a significant part of their analgesic effects with a mechanism of action based on PKC*ε* inhibition of translocation in DRG neurons.

In this paper, to elicit translocation, as well as bradykinin [12, 22], we use the peptide prokineticin 2 (PK2), involved in both inflammatory [23–25] and neuropathic [25] states, which activate PKC*ε* via Gq-coupled receptors in nociceptive neurons [16, 23]. We show with a well-established immunocytochemistry technique that gabapentin significantly and dose-dependently inhibits PKC*ε* translocation induced by bradykinin and PK2, with an effect that adds up to the effect of paracetamol.

## 2. Materials and Methods

### 2.1. Dorsal Root Ganglion Primary Cultures

Sprague-Dawley rats (2–6 weeks old) were sacrificed under total anaesthesia according to Italian and European legislation, with protocols in agreement with the guidelines of the Committee for Research and Ethical Issues of IASP published in PAIN®, 16 (1983) pp. 109-110. Experimental protocols were also approved by local institutional animal care and use committee. Dorsal root ganglia (DRGs) were collected, incubated for 1 h at 37°C with 0.125% collagenase (Sigma-Aldrich, Milan, IT), mechanically dissociated, and then plated onto glass coverslips precoated with 10 *μ*g/mL poly-L-lysine and 10 *μ*g/mL laminin (Sigma). Cells were cultured in DMEM in the presence of 1.5 *μ*g/mL cytosine 1-D-arabinofuranoside (ARA-C, from Sigma), 10% fetal bovine serum, 1% penicillin/streptomycin, and 1% L-glutamine (Invitrogen, San Diego, CA), and 100 ng/mL NGF (7s, Sigma) was also added as described previously, in order to improve neuronal survival and bradykinin and PK2 receptor expression, as it is common practice with this preparation [23, 26, 27].

### 2.2. Immunocytochemistry

Translocation of PKC*ε* from the cytoplasm to the plasma membrane was visualized as previously described [12, 19, 23]. In brief, rat DRG neurons cultured for 2 days were rapidly exposed to bradykinin at 1 *μ*M concentration or to PK2 (100 nM) for 30 s and fixed for 10 min at room temperature with paraformaldehyde (4% formaldehyde and 4% sucrose, in PBS/distilled water 2 : 1). Cells were pretreated for different times and different concentrations of gabapentin (see Results) alone or in combination with paracetamol at different concentrations (see Results). Water was used to prepare stock solutions for gabapentin, while DMSO was used for paracetamol. Final concentration of DMSO applied to cells was 1 : 1000, equal in all experiments. The stimulation with PK2 or bradykinin contained the same concentrations of drugs preapplied. Fixed cells were washed three times in PBS (with 0.1% fish skin gelatin to block nonspecific sites), permeabilized for 30 min at room temperature with Triton X-100 (0.2% in PBS with no divalent ions added), and incubated overnight at 4°C with a polyclonal anti-PKC*ε* antibody [16] diluted 1 : 1000 in PBS-T/gelatin (PBS with 0.05% Triton X-100). Coverslips were rinsed several times with PBS and then stained overnight with donkey anti-rabbit IgG conjugated to the fluorophore Alexa Fluor 488 (1 : 1000, Thermo Scientific, Waltham, MA, USA). Finally, the secondary antibody was rinsed off with PBS and cells were analyzed using a confocal microscope (Leica SP2, Leica Microsystems, Milan, Italy). Activation of PKC*ε* following activation of bradykinin or prokineticin receptors (PKRs) resulted in translocation from the cytoplasm to the neuronal cell membrane, as shown several times before [19, 20]. Translocation was quantified by determining fluorescence intensity along a line positioned across the cell in order to avoid the nucleus (for details, see Cesare et al. [21]) using semiautomated proprietary software which significantly improved efficiency of data analysis. Neurons in which intensity at the cell membrane was at least 2.0x greater than the mean of cytoplasmic intensity were counted as positive. In order to improve data reproducibility and signal/noise ratio, a very large number of cells were counted: >1000 cells per coverslip, at least 3-4 coverslips per culture, 3–12 cultures per data point. All experiments were analyzed in blind conditions.

### 2.3. Statistical Analysis

Data were analyzed by one-way analysis of variance (ANOVA), followed by Bonferroni's *t*-test for multiple comparison. An effect was determined to be significant if the *P* value was below 0.05.

## 3. Results

**Figure 1:**
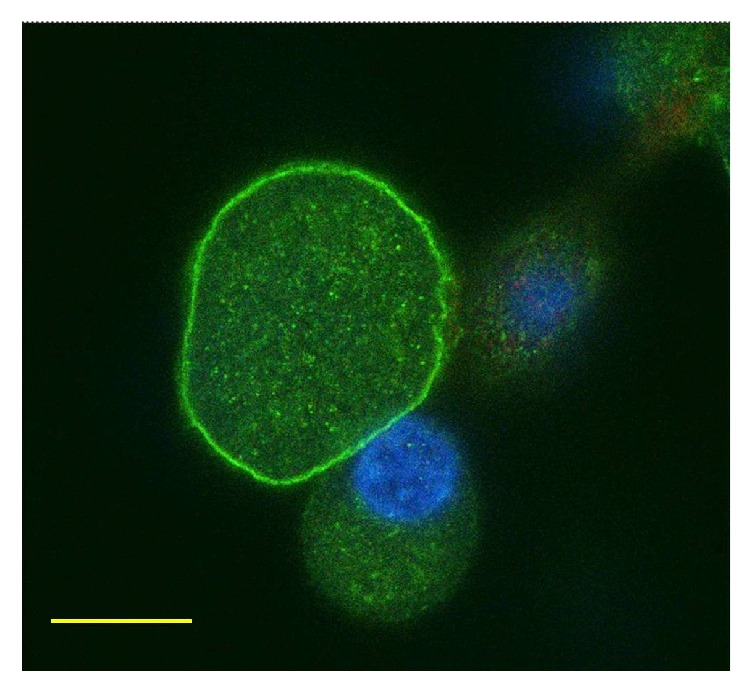
Confocal optical sections of cultured sensory neurons treated with prokineticin 2 (100 nM) for 30 seconds and subsequently fixed and stained for PKCɛ with a polyclonal specific antibody. Nuclei were stained in blue with DAPI. The largest neuron is showing translocation of PKCɛ to the plasma membrane; the smaller neurons represent a typical example of nontranslocated neurons. Translocation induced by 1 *μ*M bradykinin (BK) for 30 seconds had identical appearance. Scale bar: 5 *μ*m.

PKC*ε* activation was quantified in an all-or-nothing fashion as the number of neurons in which translocation is observed (see Figure 1), in a very large number of neurons per each experiment, in order to enhance the resolution of this technique (see Methods). Using this experimental approach, it is possible to quantitate the effect of drugs interfering with translocation and to obtain dose-response and time-course curves [23]. Following application of 1 *μ*M bradykinin or 100 nM prokineticin 2, which are saturating concentrations for these agonists, maximum translocation was consistently observed. Longer application times were avoided as they would cause PKC*ε* to be internalized, as shown and discussed previously [16, 19, 20, 23, 28]. In this set of experiments, bradykinin at 30 s produced translocation in 32.0 ± 0.6% and PK2 in 23.6 ± 0.5% of DRG neurons, consistently with previous work [12, 16, 19, 20, 26].

**Figure 2:**
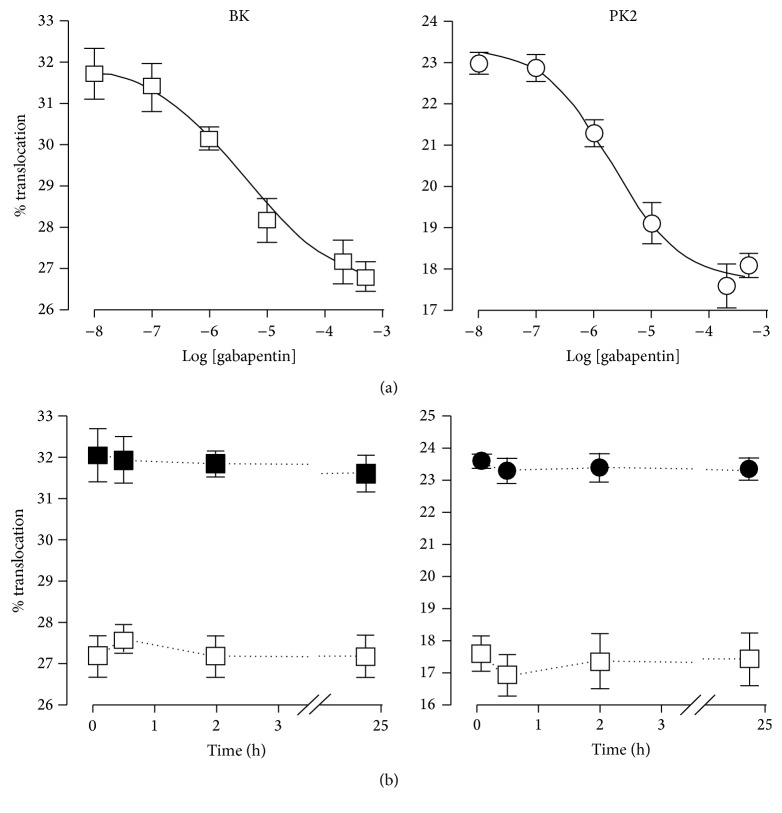
PKC translocation induced by bradykinin and PK2 is dose-dependently inhibited by gabapentin. (a) Gabapentin dose-response data on bradykinin-induced (white square symbols) and PK2-induced translocation (white circles). Gabapentin was preapplied for 5 minutes, and then neurons were treated with 1 *μ*M bradykinin or 100 nM PK2 for 30 s in the presence of the same preapplied concentration. (b) Time course of translocation at different times in control culture medium (black symbols) and in the same medium with 200 *μ*M gabapentin added (white symbols). Both bradykinin- and PK2-induced translocation and gabapentin effect are not time-dependent in these conditions. See Results for further details. Notes: values are means ± SEM of data from 4–7 separate cultures.

As shown in Figure 2(a), the percentage of neurons in which bradykinin receptor activation caused translocation was dose-dependently decreased by 5 min preapplication of gabapentin in the range 10 nM–500 *μ*M to a value of 26.8 ± 0.7% for the largest concentration tested. Inhibition therefore was about 17.8%. Similarly, the control value of translocation for PK2 experiments decreased to 17.6 ± 0.5 for the concentration of 200 *μ*M gabapentin, with overall inhibition of about 34%.

Dose-response curves were well fitted with the Hill equation with the following parameters: for bradykinin-induced translocation, gabapentin ED_50_ = 3.8 ± 0.7 *μ*M and Hill slope = 0.5 ± 0.1; for PK2-induced translocation, ED_50_ = 1.9 ± 0.3 *μ*M and Hill slope = 0.7 ± 0.1.

Dose responses were obtained after 5 min pretreatment with gabapentin, applied in culture medium and present also in the bradykinin or PK2 stimulation solution. In Figure 2(b), we show that longer treatments (30 min, 2 hours, and 24 hours) produced no significant time-dependent change in the fractional effect of gabapentin at 200 *μ*M concentration.

**Figure 3:**
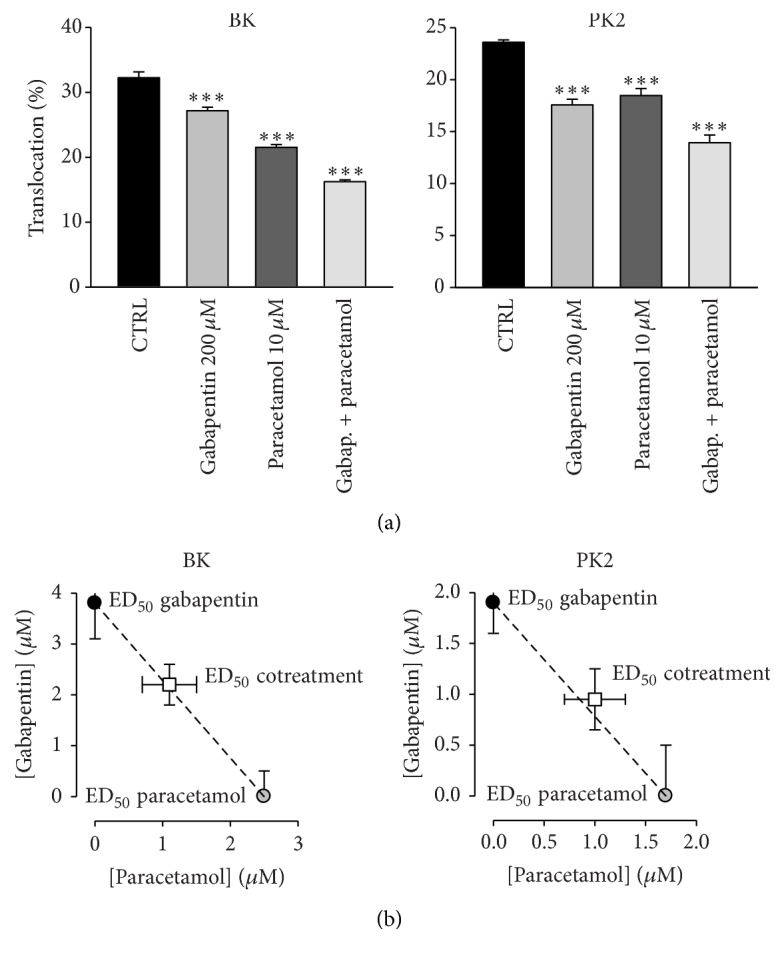
PKC translocation suppression by gabapentin and by paracetamol is additive. (a) Experiments on bradykinin- (BK-) induced and PK2-induced PKC*ε* translocation. Gabapentin effect on the latter was almost double compared to the former, with about 14.2% suppression on bradykinin-induced translocation and 25.4% suppression on PK2-induced translocation, compared to control (CTRL). Paracetamol-induced suppression was 33.4% (BK) and 21.6% (PK2). Combined drug suppression was, respectively, 49.8% and 41.9%, consistent with an additive effect of these drugs. (b) Isobolograms for the effects of gabapentin and paracetamol, alone or in combination, in BK- and PK2-induced PKC*ε* translocation. White square symbols correspond to the experimental cotreatment ED_50_ with 95% confidence limits. Circle symbols correspond to ED_50_ for gabapentin and paracetamol alone. Notes: values are means ± SEM of data from 5 separate cultures. ^*∗∗∗*^*P* < 0.001 versus control and versus other treatments in all combinations (ANOVA followed by Bonferroni's *t*-test).

As described in Figure 3(a), paracetamol (10 *μ*M), as previously observed [19, 20], inhibited translocation by bradykinin. In this new set of experiments, translocation was reduced from the control value of 32.3 ± 0.9 to 21.5 ± 0.4%. A similar result was obtained with PK2-induced translocation from 23.6 ± 0.2% to 19.0 ± 0.5%. All experiments were repeated in 5 different cultures.

Combined application of gabapentin and paracetamol resulted in summation of the respective effects, with a reduction from the control value to 16.2 ± 0.3% and to 13.2 ± 0.4%, respectively, in experiments with bradykinin and with PK2.

In order to further investigate the nature of the apparently additive behavior of gabapentin and paracetamol effect, we performed a full isobolographic analysis, which confirmed an additive behavior, with no sign of synergistic interaction (Figure 3(b)).

## 4. Discussion

Gabapentin, paracetamol, nonsteroid anti-inflammatory drugs (NSAIDs), selective COX-2 inhibitors, and glucocorticoids are nonopioid drugs which display clinically relevant analgesic properties. In postoperative pain management, patients are very often treated with combinations of the above nonopioid analgesics to reduce opioid-related adverse effects. Although a variety of combinations have recently been tested and although some of them are currently employed in clinical practice [29], the rationale and the molecular mechanisms behind the combined use of nonopioid analgesic drugs are often not quite well documented. Recently, it was shown that gabapentin in combination with other drugs with minimal adverse effects such as paracetamol [30] allowed significant opioid sparing with positive effects in postsurgical patients. So far, gabapentin was believed to produce its antinociceptive effects mainly by actions on voltage-gated ion channels, neurotransmitter ionotropic receptors, and L-amino acid transporters [31]. Such actions are therefore supposed to occur centrally, where most of such sites of action are located. Indeed, gabapentin actions both in spinal and in supraspinal sites have been described [32]. Previously, some involvement of PKC in antinociceptive effects of gabapentin was reported in the trigeminal nucleus [33] and in spinal cord dorsal horn [34]. In a very recent report [35], this result was confirmed, with indirect evidence of the involvement of dorsal horn *ε* and *γ* PKC isoforms in a visceral pain model [36]. The western blot technique employed in those studies does not allow discrimination of the cell localization where PKC*ε* inhibition of translocation occurs, but a contribution of presynaptic PKC*ε*, present in the central terminal of the sensory neuron, consistent with the one we report here, is largely possible. The *γ* isoform of PKC (PKC*γ*) is not expressed in dorsal root ganglia [16]; therefore, activation of PKC*γ* can only occur downstream of the sensory neuron, or in surrounding nonneuronal cells. In this context, our paper contributes the first demonstration of PKC-involving antinociceptive functions of gabapentin in the therapeutic range of concentrations in the peripheral nervous system, by providing the first direct evidence that a dose-dependent, significant effect is produced by gabapentin with a mechanism that involves specific inhibition of translocation of the *ε* PKC isoform in sensory neurons. This mechanism is likely a relevant part of gabapentin antinociceptive action, as the lack of translocation will prevent sensitization of membrane targets in the significant percentage of sensory neurons where complete inhibition occurs.

The present study shows also that the effect of gabapentin combined with paracetamol, in comparison to each drug alone, adds to the effect of each other. This observation is consistent with a previous study on acute postoperative pain, demonstrating that the combination of gabapentin and paracetamol reduced morphine self-administration in a randomized and double-blinded manner in human patients suffering from postoperative pain [6]. Although in that study it is not possible to discriminate between peripheral and central effects of these drugs, our study suggests that the peripheral effect is likely to contribute significantly to the overall analgesia. In this context, the observed inhibition of PKC*ε* translocation by gabapentin and by paracetamol appears to be highly relevant for a proper understanding of the multifactorial pharmacological actions of these drugs. Inhibitory effects by gabapentin and paracetamol were largely similar both in bradykinin- and in PK2-induced translocation, suggesting that these drugs do not specifically interact with bradykinin and PK2 membrane receptors but rather affect mechanisms of translocation intracellular pathway, possibly directly on PKC*ε* itself, or on the interaction of PKC*ε* with the receptors for the specific activated protein kinase C isoform, RACKs [37]. The fact that gabapentin and paracetamol effects on PKC*ε* are additive suggests that the overall inhibition of translocation is likely obtained with one or perhaps two different molecular mechanisms activated by each drug, converging on the same downstream action on PKC*ε*, which are present in specific subsets of neurons. Sensory neurons are well known to represent a highly heterogeneous population, with different morphological, neurochemical, and biochemical properties [38]. The observation that the gabapentin-induced reduction of fractional suppression of translocated neurons is about double for PK2-stimulated in comparison to bradykinin-stimulated neurons (see Results) is consistent with the difference in populations expressing the PK2 receptor, the latter almost exclusively isolectin B4-negative [23] and the former both isolectin B4-positive and isolectin B4-negative (unpublished observation, consistent with the literature [12, 39, 40]) only partially overlapping [23].

More work is necessary to determine the nature of mechanisms preventing translocation in these specific subsets of neurons. Further experiments that will determine whether other novel or classic antinociceptive drugs currently in use, as well as paracetamol and gabapentin, produce part of their effect via inhibition of PKC*ε* translocation and whether their combined effects are additive or possibly synergistic are currently in progress in our labs.

## 5. Conclusion

In conclusion, our results help to clarify the pharmacological action of largely used drugs such as gabapentin and paracetamol and of their combined use. From these results, in combination with data in the literature, gabapentin emerges as a drug with multifactorial modes of action on the pain pathway, with an increasing number of novel, interesting mechanisms not only in the central but also in the peripheral nervous system.
